# The extent of visual space inferred from perspective angles

**DOI:** 10.1068/i0673

**Published:** 2015-01-06

**Authors:** Casper J. Erkelens

**Affiliations:** Helmholtz Institute, Utrecht University, Utrecht, The Netherlands; e-mail: c.j.erkelens@uu.nl

**Keywords:** Euclidean geometry, perspective angles, slant, vanishing points, visual space

## Abstract

Retinal images are perspective projections of the visual environment. Perspective projections do not explain why we perceive perspective in 3-D space. Analysis of underlying spatial transformations shows that visual space is a perspective transformation of physical space if parallel lines in physical space vanish at finite distance in visual space. Perspective angles, i.e., the angle perceived between parallel lines in physical space, were estimated for rails of a straight railway track. Perspective angles were also estimated from pictures taken from the same point of view. Perspective angles between rails ranged from 27% to 83% of their angular size in the retinal image. Perspective angles prescribe the distance of vanishing points of visual space. All computed distances were shorter than 6 m. The shallow depth of a hypothetical space inferred from perspective angles does not match the depth of visual space, as it is perceived. Incongruity between the perceived shape of a railway line on the one hand and the experienced ratio between width and length of the line on the other hand is huge, but apparently so unobtrusive that it has remained unnoticed. The incompatibility between perspective angles and perceived distances casts doubt on evidence for a curved visual space that has been presented in the literature and was obtained from combining judgments of distances and angles with physical positions.

## 1 Introduction

Visual space is the space that we perceive as the consequence of instantaneous visual stimulation of the eyes. The structure of visual space has been the subject of many experimental, theoretical, and philosophical studies (Blank, 1961; Blumenfeld, 1913; Cuijpers, Kappers & Koenderink, 2000; Foley, 1972; Hatfield, 2003; Higashiyama, 1984; Hillebrand, 1902; Indow, 1991; Koenderink, van Doorn, & Lappin, 2000; Luneburg, 1950; Musatov, 1976; Suppes, 1977; Todd, Oomes, Koenderink, & Kappers, 2001; Wagner, 1985). Most studies have been concerned with the near-binocular space. The properties of a richly structured visual space beyond near distance have received less attention. Recent studies have shown that properties of the ground surface in the real world can significantly affect distance perception (Sinai, Ooi, & He, 1998; Wu, Ooi, & He, 2004). Distance estimation was found to be very accurate for flat and continuous surfaces up to 20 m (Sinai et al., 1998).

In a way it is remarkable that far visual space has received little attention because objects and structures in scenes show that far visual space has interesting features different from physical space. From just looking at a straight railway line or road, it is obvious that visual space contains perspective properties similar to linear perspective in 2-D pictures. Euclid called the perspective nature of vision natural perspective (Koenderink, 2012). Actually, it is odd that visual space is such a deformed representation of physical space. In view of plasticity of cortical maps (Singer, 1995), one would expect that visual space would adapt to long-term and systematic deviations from physical space. Apparently, adaptation does not occur. Instead, human beings have both perspective and Euclidean representations of physical space at their disposal. For example, we see on the one hand that a road narrows in front of us but on the other hand we are confident that it does not. The availability of different representations gives human beings the possibility to answer questions about spatial relationships in different ways. For instance: What is the angle between rails of a railway track? One answer may reflect the perspective projection of the rails on the retina whereas the other may express experience with natural perspective in general or knowledge of rails in particular. The purpose of this study is to explore perspective properties of visual space.

**Figure 1.**
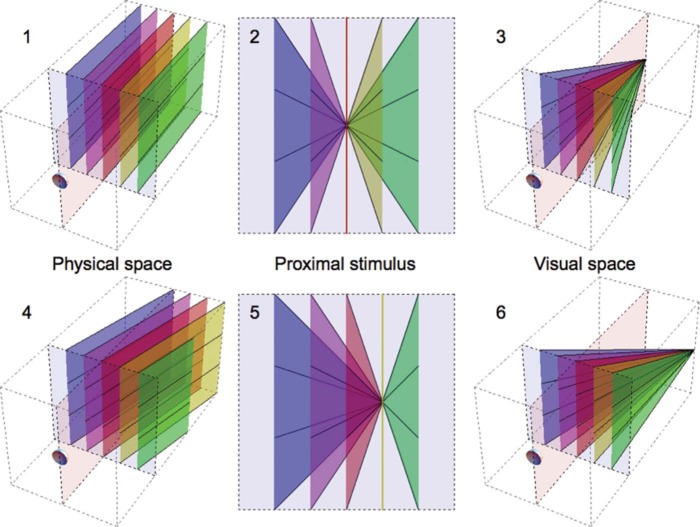
Spatial transformations. Left figures: Parallel lines and planes in physical space run from a screen (light blue) in front of an eye to infinity (just a finite part is shown). Center figures: Projections of the lines and planes on the frontal screen represent the proximal stimulus. Right figures: Visual space is postulated to be the 3-D expansion of the proximal stimulus in depth limited to a finite vanishing point. The vanishing point is in the straight-ahead direction in the top figures and in a rightward direction in the bottom figures. The light-red vertical plane indicates the sagittal plane.

In vision, two processes may have a significant impact on the representation of the physical space. One process is optical and the other neural of nature. In Figure 1, sets of lines and planes are drawn to show how the two processes retain and change specific spatial properties. The first process is the optical transformation from 3-D to 2-D space and occurs in the eye where light reflected from objects in physical space is projected onto the retina. In Figure 1, the retinal projection, i.e., proximal stimulus, is imaged as a pattern on a screen placed in front of the eye perpendicular to the straight-ahead direction. The projection on the screen is a planar representation of the retinal stimulus. The proximal stimulus is inevitably a perspective projection of physical space because the eye collects spatial information from a single vantage point. A consequence of linear perspective is that straight lines in the proximal stimulus are projections of straight lines in physical space (Tyler, 2014). Another consequence of linear perspective is that projections of lines of one orientation in physical space (1 in Figure 1) converge to a single point in the proximal stimulus (2 in Figure 1). Lines of different orientation (4 in Figure 1) have a different point of convergence (5 in Figure 1).

The second process, occurring in the brain, assigns depth to the proximal stimulus. As a consequence of the two consecutive transformations, locations in 3-D physical space are represented as combinations of visual directions (2-D) and depth (1-D) in visual space. Information about visual directions follows directly from retinal and eye position signals whereas depth requires interpretation of the proximal stimulus (Erkelens, 2012). A perspective visual space is not the inevitable consequence of the perspective nature of the proximal stimulus. If the brain would assign veridical depth to each visual direction, visual space would be a faithful Euclidean, and thus nonperspective, representation of physical space (1 in Figure 1). In such a visual space, vanishing points would be at infinite distance from the viewer. As mentioned before, it is evident that human visual space shows perspective properties. A way to transfer the proximal stimulus into such a visual space is to assume that visual vanishing points are positioned at finite depth (3 and 6 in Figure 1). The idea of a finite visual space is not new. Already, James (1890) and later Luneburg (1947) proposed the concept. Gilinsky (1951) elaborated the consequence of finite vanishing points for perception of distance. The consequences of a visual space having vanishing points at finite distance, here dubbed as perspective visual space, have not yet been explored for slant perception.

**Figure 2.**
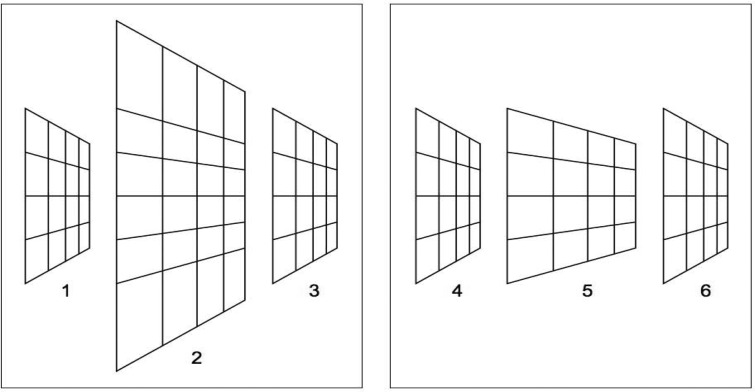
Slanted grids. Grids 1, 3, 4, and 6 are identical of shape. One obtains the impression that each of these grids is slanted differently. This phenomenon is known as the Leaning Tower illusion. Grid 5 is identical to the middle part of grid 2. The lines of grids 2 and 3 converge to a single vanishing point, as do the lines of grids 5 and 6. Grids 2 and 3 appear aligned but grids 5 and 6 do not.

Figure 2 demonstrates that a perspective visual space is attractive for understanding slant perception. The small grids 1, 3, 4, and 6 are identical in shape. However, due to their horizontal position in the figure, the lines of the grids converge to different vanishing points. Perceptually, the slants of the grids differ in that the slant appears to decrease from left to right. This difference in perceived slant is a demonstration of the Leaning Tower illusion (Kingdom, Yoonessi, & Gheorghiu, 2007). Figure 1 shows that the illusion is consistent with a perspective visual space. For instance, the magenta triangle in proximal stimulus 2 and the red triangle in 5 are horizontally shifted identical shapes. The visual spaces 3 and 6 show that the triangles will be perceived as differently slanted triangles in 3-D space. Figure 2 shows another, novel, illusion that is consistent with a perspective visual space but not with a Euclidean one. Grids 2 and 5 are different in size and shape. However, grid 5 is identical to the middle part of grid 2. The lines of grids 2 and 3 converge to a single vanishing point, as do the lines of grids 5 and 6. Grids 2 and 3 appear to slant in one direction as if the grids form a single, planar surface, grid 2 appearing nearer than grid 3. Grids 5 and 6 are seen at about equal depths, and grid 5 appears much less slanted than grids 2 and 6. The different slants and depths of grids 2 and 5 can be understood in terms of the perspective geometry of visual space. If visual space would be Euclidean, grids 2 and 5 of Figure 2 would be perceived as parallel grids at different depths. As can be observed in visual space 6 of Figure 1, there is a relationship between perceived slant and depth in perspective visual space. Along a visual direction, nearer surfaces are more slanted. In the sagittal plane of visual space 6 of Figure 1, as an example, the red surface is nearer and more slanted than the magenta surface which, in turn, is nearer and more slanted than the blue surface. Perceived slants and depths of grids 2 and 5 of Figure 2 show the same relationship.

The grids of Figure 2 are pictures and not real objects in physical space and because of this, perception of these grids takes place in the realm of pictorial rather than visual space. Studies of perceived deformations during oblique viewing of pictures have suggested that pictorial space is different from visual space (e.g. see Cutting, 1987; Koenderink & van Doorn, 2012; Kubovy, 1986). Several studies have presented evidence that the visual system exploits information about the slant of the picture surface itself to compensate for viewing angle (Goldstein, 1987, 1988; Halloran, 1993; Perkins, 1973; Rosinski, Mulholland, Degelman, & Farber, 1980; Vishwanath, Girshick, & Banks, 2005; Wallach & Marshall, 1986). Other authors have found no evidence for any correction mechanism (Koenderink, van Doorn, Kappers, & Todd, 2004; Rogers & Gyani, 2010). In two recent studies, experiments and computations showed that the perceived slant of pictured grids viewed from oblique directions was explained by linear perspective and Euclidean geometry (Erkelens, 2013a, 2013b). Apart from a stronger underestimation of slant, there was no reason to assume a different geometry for pictorial space.

**Figure 3.**
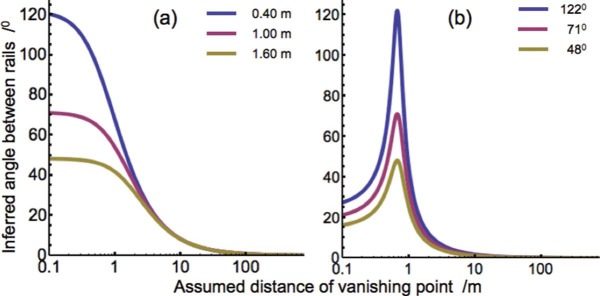
Computed perspective angles. **A.** Inferred angles between rails of a railway line as a function of the assumed distance of the vanishing point for three heights of the eye above the track. **B.** Inferred angles between the rails viewed in pictures (see Figure 4) as a function of the assumed distance of the vanishing point. The screen was positioned 0.57 m in front of the observer. The near ends of the rails were assumed to lie on the screen.

The concept of a perspective visual space makes predictions for perspective angles between parallel lines in physical space than run away from the observer. The visually perceived angles should be larger than zero and smaller than the angles between projections of the physical lines in the proximal stimuli. Computations were made for an observer standing between rails of a straight railway track (stimulus geometry and equations are presented in the Appendix). The track gauge, i.e., the distance between the rails, was 1.435 m. Angles between the rails were computed for three heights of the eye above the track as a function of the distance of the vanishing point (Figure 3A). For a distance of 0 m, the rails constitute a triangle whose vertex angle measures 122°, 71°, and 48° for eye heights of 0.4, 1.0, and 1.6 m, respectively. The perspective angles between the rails reach these values in the proximal stimulus (see Appendix). Figure 3A shows that the angles are reverse sigmoid functions of the distance of the vanishing point. For a distance of 10 m, the perspective angle is reduced to about 8° for all the three eye heights. For a distance of the vanishing point of 100 m, the angle is already as small as about 0.8°. To compare distances of vanishing points in visual and pictorial space, pictures of the track were taken from the various positions of the eye and displayed on a frontal screen placed at such a distance from the observer that the pictures were viewed from the center of projection. Screen positions of the near and far ends of the rails were used to compute perspective angles (Appendix). The near ends were kept fixed on the screen. The location of the far end was varied along the line running through the center of projection and the far end of the rails on the screen. Figure 3B shows that in pictorial space perspective angles have peak values at the distance of the screen corresponding to the values of the angles in the proximal stimuli. Due to the limited field of view of the screen and the choice to keep the near ends of the rails fixed on the screen, perspective angles decrease faster with distance in pictorial space than visual space. Insight in the distances of vanishing points of the visual and pictorial spaces of human observers was obtained by measuring how they judged perspective angles between physical as well as depicted rails.

**Figure 4.**
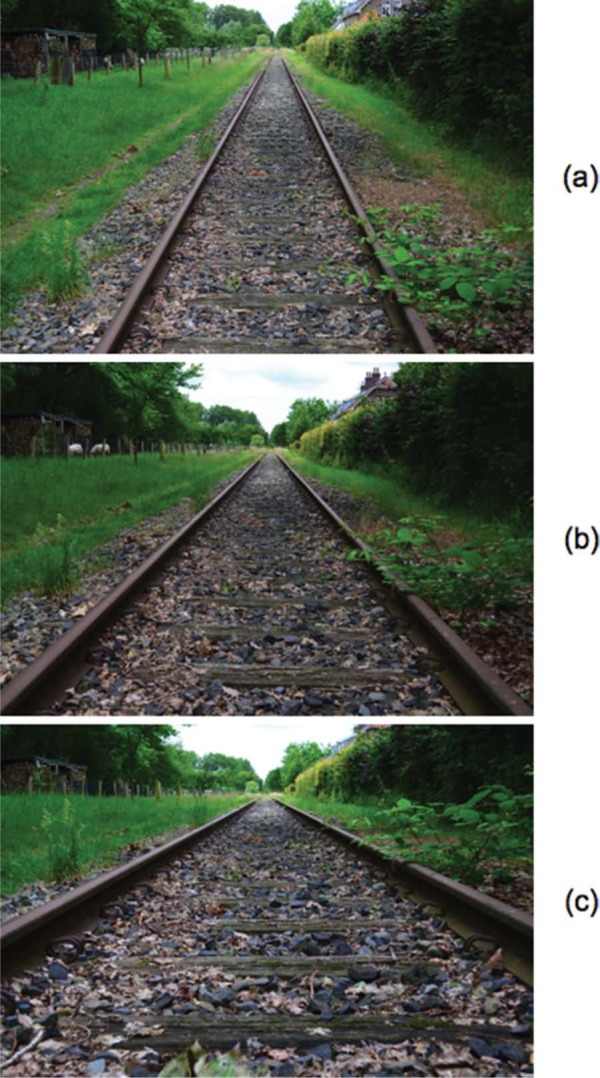
Disused railway track. The angle between the rails was judged at three different eye heights, 1.60 m (a), 1.00 m (b), and 0.40 m (c), respectively. The angles between the rails were 48° (a), 71° (b), and 122° (c) in the proximal stimuli.

## 2 Experiment

To measure perspective angles between rails, a disused railway track was chosen between the villages Boxtel and Veghel in the province of Noord Brabant in The Netherlands. A straight section of the track, located between Gemondestraat and Savendonksestraat at Liempde, was found suitable for the task (Figure 4).

### 2.1 Stimuli and experimental setup

A pair of compasses was used to indicate the perspective angle of the rails. Judgments in the field were made at three eye heights of 1.6, 1.0, and 0.4 m, respectively. From the same positions, pictures were taken with a Nikon D5100 camera fitted with a normal prime lens (Nikkor DX AF-S 35mm f/1.8 G). A normal lens was chosen because it produces perspective in pictures that, if viewed from the correct distance, is natural to a human observer (Cooper, Piazza & Banks, 2012). Field of view of the camera–lens combination was approximately 38° × 26°. The pictures were used to compare perspective angles of physical and depicted rails mediated by similar proximal stimuli. The pictures were displayed on a TFT monitor (21″ LaCie 321, 1600 × 1200 pixels, 75 Hz). The screen measured approximately 43° × 28° at the viewing distance of 0.57 m. The pictures were projected at a size that was identical to the field of view of the camera–lens combination. A chin rest was used to fixate head position so that the center of the forehead (the “cyclopean eye”) was positioned at the center of projection of the pictures. The setup was placed in a normally lit room.

### 2.2 Procedure

Three subjects (two physics students and the author) judged angles in the field as well as the laboratory. The two students were experienced with judging angles in previous slant experiments but were naive with respect to the purpose of the study. The subjects had normal or corrected-to-normal vision and gave informed consent in accordance with the Declaration of Helsinki. The subjects' eye height was measured and adjusted when needed before they made their judgments on the railway track. To allow the three different eye heights, they had to stand, sit on their knees, and lie down on the track, respectively. For each measurement, the subject estimated the perspective angle between the rails, turned to the left or right, held the compass in a vertical position, and adjusted the angle between the legs until it was judged to match the remembered angle. Turns of either head or torso of almost 90° to the left or right were made to prevent the subject from seeing rails and compass in a single view. The compass was held in a vertical position so that perspective angles of the rails were matched to zero perspective angles of the compass. The subject was allowed to repeat the procedure until he was satisfied with the result. The measurements were repeated 10 times during monocular and binocular viewing. The legs of the compass were closed after each measurement. The same procedure was applied in the laboratory where the subjects judged the angles between the rails in the photographs and between lines on the screen mimicking the rails without any perspective context.

## 3 Results

**Figure 5.**
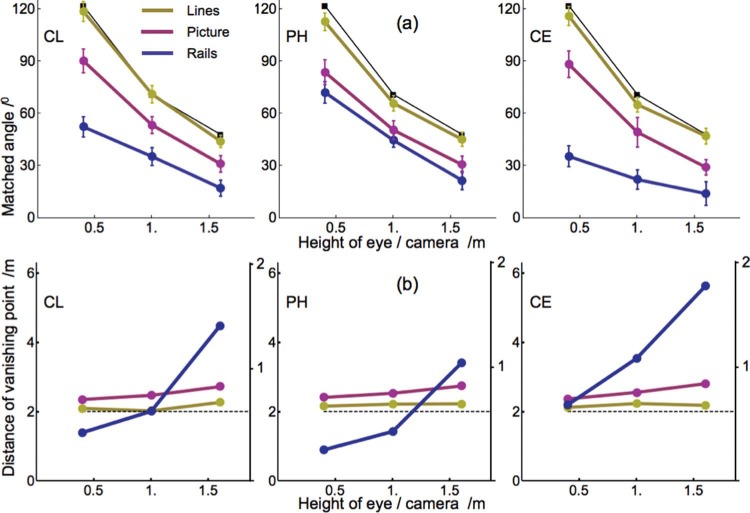
Matched angles and computed distances of vanishing points. **A.** Mean perspective angles (±1 SD) between the rails matched by the three subjects. The judgments were made for physical rails (Rails), depicted rails (Picture), and for lines on a further blank screen (Lines). The black dots indicate the angles of rails and lines measured on the screen, i.e., the proximal stimulus. **B.** Distances of the vanishing points computed from the judged angles using the inverse relationship expressed by Equation (3) of the Appendix. The left axes indicate distances for the Rails data, the right axes distances for the Picture and Lines data. The dashed lines indicate the distance of the screen relative to the viewer.

Figure 5A shows the matched angles for each individual observer as a function of height of eye or camera. The Lines data show that the subjects judged angles between lines without perspective context with small errors. Angles between rails were judged considerably smaller than between the lines, especially between physical rails. No differences were found between binocular and monocular data. Apart from a few outliers, individual data, both binocular and monocular, differed less than 10° from the mean in each condition. Rails angles ranged from 27% to 63% of the Lines angles and Picture angles from 60% to 83%. Distances of vanishing points were computed from their relationship to the perspective angle. For physical rails, the inverse relationships are shown in Figure 3A and for depicted rails in Figure 3B. The monotonous relationships of Figure 3A gave unique solutions, the peaked relationships of Figure 3B generally gave two solutions. In the case of two solutions, the distance longer than the eye-screen distance gave the appropriate distance of the vanishing point. The computed distances were very short in relation to the length of the visible track. For physical rails, the vanishing distance ranged from just 1.02 to 5.97 m. Distance increased with eye height. For depicted rails, the vanishing distance ranged from 0.79 to 0.94 m. Since the screen was 0.57 m away from the observer, depth between the far and near ends of the rails ranged just from 0.13 to 0.27 m in the three subjects.

## 4 Discussion

### 4.1 Main conclusions

Judgments of perspective angles were reproducible and consistent across the three subjects. Angles between rails were judged considerably smaller than their sizes in the proximal stimulus. Angles between physical rails were judged smaller than those between depicted rails. Perspective angles were found to represent a visual space in which visual directions and depth have very different metrics. Visual space inferred from perspective angles between rails extends less than 6 m in depth. If the inferred visual space would be congruent to physical space, i.e., Euclidean space, we would have expected perspective angles close to zero degree. If the inferred visual space would extend to depths of 100 m or more, as is generally accepted for the depth of visual space, we would have expected angles below 1°. If perspective angles would be unrelated to visual space, we would have expected to measure perspective angles similar to the angles between rails and lines in the proximal stimulus. The main experimental finding of this study is that perspective angles differed considerably from any of the expected values. The perspective angles were compatible with a visual space of a few meters and a pictorial space of just a few tenths of meters. Visual space inferred from perspective angles does not seem to have a relationship with visual space as human observers experience it. We, the three subjects, were convinced that the distance of the visually perceived vanishing point of the physical railway track was far beyond the distance computed from the perspective angles. It has also been established that human observers can accurately judge the absolute distance of objects up to 20 m when these are viewed with reference to a flat terrain (Loomis, Da Silva, Philbeck, & Fukushima, 1996; Wu et al., 2004). Our experience of visual space, however, may not just reflect perceived distances per se but include an appreciation of distance based on experience with walking along a railway track or with physical distance in general (Gilinsky, 1951).

### 4.2 Relation to other models of visual space

Perspective space as a model for visual space is interesting with respect to other proposals for visual space reported in the literature. Best known is the classic model of a Riemannian visual space as was proposed by Luneburg (1950) and tested and amended by many other authors (Blank, 1961; Cuijpers et al., 2000; Cuijpers, Kappers, & Koenderink, 2002; Foley, 1972; Higashiyama, 1984; Indow, 1991; Koenderink et al., 2000; Musatov, 1976; Wagner, 1985). Three differences between the current approach and those of previous studies are relevant for the validity and applicability of the proposed models. 1. Many visual-space studies have used isolated stimuli in nearly empty or little-structured spaces. The current study used a highly perspective, natural scene. Two studies explicitly showed that context influences visual space (Cuijpers, Kappers, & Koenderink, 2001; Schoumans, Koenderink, & Kappers, 2000). As a consequence, different models may not oppose each other but describe visual space for different environments. 2. In previous studies, stimuli were specified in visual space (e.g., “parallel,” “equidistant,” “pointing to”) and responses were geometric quantities (e.g., orientations, positions) of physical space. Such experiments may reveal the transformation from visual to physical space, although several authors have claimed that Luneburg's model describes the intrinsic geometry of visual space (Blank, 1961; Indow, 1991) or the mapping from physical to visual space (Cuijpers et al., 2002). In the current study, stimuli were a property of physical space, namely parallelism of rails, and responses were angles between rails in visual space. The current measurements examined the transformation of physical into visual space. 3. Previous studies used properties (e.g., “parallel,” “collinear”) that were not defined in visual space for as long as the geometry of visual space was unknown. As a result, it seems not justified that these properties were used to explore the geometry of visual space. An assumption in the current study was that it was deemed justified to compare perspective with nonperspective angles.

The visualization of visual space as a flat space (Figure 2) is in conflict with studies claiming that visual space is curved. An important conclusion of the current study is that perspective angles and distances do not merge into a single visual space. Perceived angles and distances, however, have been elements for the construction of curved visual spaces. Judgments of angles and distances in parallel and distant alley tasks (Blank, 1961; Blumenfeld, 1913; Hillebrand, 1902; Luneburg, 1950), an exocentric pointing task (Koenderink et al., 2000), and a bisection task (Todd et al., 2001) were combined with physical positions and distances of stimuli to construct visual spaces. The incompatibility between perspective angles and perceived and physical distances casts doubt on the validity of claims for a curved visual space.

### 4.3 Parallelism and collinearity

Parallelism and collinearity are related properties of metric spaces. Collinearity is a special case of parallelism in Euclidean as well as Riemannian spaces. Cuijpers et al. (2000, 2002) tested the Riemannian nature of visual space by comparing settings of bars in “parallel” and “collinear” tasks. Cuijpers et al. (2002, 2002) found large deviations, measured in Euclidean physical space, from parallel in the “parallel” task and small deviations in the “collinear” task. The large deviations in the “parallel” task were interpreted as evidence that visual space was not Euclidean and the small deviations in the “collinear” task as evidence that visual space was Riemannian neither. The perspective visual space proposed here describes the observed deviations if human beings use parallelism and collinearity as defined in Euclidean space. Figure 1 shows that lines are not parallel in physical and visual space at the same time. Parallel lines in physical space converge in visual space and, thus, parallel lines in visual space diverge in physical space. This means that if bars are set parallel in visual space, the bars diverge in physical space. Indeed, Cuijpers et al. (2002, 2002) measured large diverging deviations in “parallel” tasks. Figure 1 also shows that collinear lines in physical space are collinear in visual space and vice versa. The bars have different orientations in physical and visual space. However, this difference does not affect their collinearity. Thus, if bars are set collinear in visual space, the bars are collinear in physical space. This prediction is in agreement with the results of Cuijpers et al. (2002, 2002).

### 4.4 Doubt about a single visual space

The current measurements and computations show that perspective angles and depth are not integrated into a coherent visual space. The perspective angles of rails are consistent with a visual space that is just a few meters deep whereas experienced depth seems to extend over hundreds of meters. The incongruity between perspective angles and depth is apparently so unobtrusive that it has remained unnoticed until now.

Distances of vanishing points inferred from perspective angles were considerably different for the visual and pictorial spaces. There are a few possible explanations. The reduced depth of pictorial space may be related to the strong underestimation of slant that has been measured for depicted grids viewed from oblique directions (Erkelens, 2013a, 2013b). The most probable explanation given in those studies was that the effectiveness of perspective information was suboptimal (Erkelens, 2013a). Another possibility is that cues to slant and depth oppose each other in pictorial space and support each other in visual space. The reduced depth of pictorial space may also be caused by an assumption underlying the computations of distance of vanishing points. Computations were based on the assumption that nearest parts of the depicted rails are seen at the distance of the picture. If the assumption is false, differences in extents of the pictorial and visual spaces may be fictitious.

As has been argued before, incompatibility between perspective-angle inferred depth and experienced depth casts doubt on the existence of a visual space that is consistent for distances and angles. Visual space may be a practical vehicle for describing perceptual responses to local stimuli in a global framework, but not much else. Previous studies showed that visual space also depends on task (Blumenfeld, 1913; Cuijpers et al., 2002; Hillebrand, 1902), context (Schoumans et al., 2000), and reference frames (Cuijpers et al., 2001). The current analysis suggests that vision occurs in a Euclidean space where for instance parallelism and collinearity are well-defined properties. Viewing the world from a vantage point, the commonly used argument for seeing a distorted physical space, does not preclude a Euclidean visual space. The combination of a vantage point and underestimation of depth causes the perspective appearances of objects and scenes. Common geometries of physical and visual spaces, one is Euclidean and the others are linear transformations, may explain why adaptation of visual space does not reduce long-term perspective distortions. It does not explain why visual space inferred from perspective angles is so extremely shallow.

## Figures and Tables

**Figure 6.**
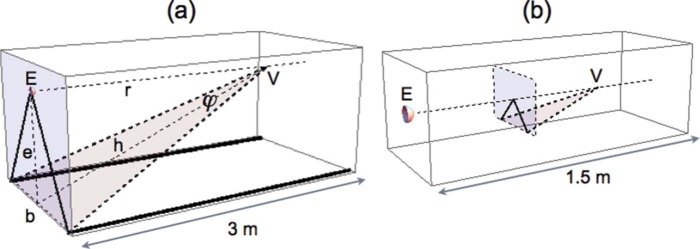
Stimulus geometry. (a) The subject viewed (E represents the position of the eyes) physical rails (thick black lines) from a central position between the rails. The rails seemed to converge to vanishing point V. (b) The subject viewed rails depicted on the vertical screen (blue). The relationship between perspective angle *φ* at V and distance of the vanishing point (*r*) was computed from the geometry of the isosceles triangle (red) with base *b* and height *h*, and eye height *e*. The proximal stimuli (projections in the blue planes) are identical in (a) and (b). For reasons of clarity the examples (a) and (b) are fictitious and are drawn at different scales.

## Appendix

The relationship between perspective angle (*φ*) and distance between viewer and vanishing point (*r*) is illustrated in Figure 6 for viewing disappearing rails of a real railway line (a) and for viewing the same rails in a picture (b). The relationship is determined by two equations. Trigonometry of the red isosceles triangle of Figure 6a provides one equation, namely,

(1) $$\phi =2\arctan\left(\frac{b}{2h}\right)$$

and the Pythagorean theorem provides the other,

(2) $$e^{2}+r^{2}=h^{2}.$$

Elimination of *h* from equations 1 and 2 gives

(3) $$\phi =2\arctan\left(\frac{b}{2\sqrt{e^{2}+r^{2}}}\right).$$

Relationship (3) also holds for the perspective angle shown in Figure 6b if e is replaced by height of the rails in the picture and r represents the distance between screen and vanishing point.
